# Long working hours, job satisfaction, and depressive symptoms: a community-based cross-sectional study among Japanese employees in small- and medium-scale businesses

**DOI:** 10.18632/oncotarget.18084

**Published:** 2017-05-23

**Authors:** Akinori Nakata

**Affiliations:** ^1^ School of Health Sciences, University of Occupational and Environmental Health, Kitakyushu, Japan

**Keywords:** working hours, job satisfaction, depressive symptoms, work condition, Japan

## Abstract

Although long working hours have been suspected to be a risk factor for depressive symptoms (DS), it is not well understood the conditions under which long working hours are associated with it. This study investigated the moderating effect of job satisfaction on the relationship between working hours and DS. A total of 2,375 full-time non-shift day workers (73% men), aged 18–79 (mean 45) years, in 296 small- and medium-scale businesses were surveyed using a self-administered questionnaire evaluating working hours, job satisfaction, DS and covariates. The Center for Epidemiologic Studies Depression scale (CES-D) was used to assess DS. Risk of DS (CES-D ≥ 16) by working hours, job satisfaction, and both combined was estimated by multivariable logistic regression analysis. Compared to participants working 6–8 hrs/day, those working 12+ hrs/day had significantly higher odds of DS (adjusted odds ratio [aOR] 1.49), while participants with low satisfaction, as opposed to high satisfaction, had increased odds of DS (aOR 1.81). Furthermore, compared to those working 6–8 hrs/day with high satisfaction (reference group), participants working 6-8 hrs/day, > 8 to 10 hrs/day, and > 10 hrs/day combined with low satisfaction had dose-response increase of DS (aOR 1.48, 2.21 and 2.31, respectively, *p* < 0.05), whereas those working > 8 to 10 hrs/day and > 10 hrs/day combined with high satisfaction had not (aOR 0.93 and 1.39, respectively, *p* > 0.10). The results suggest that long working hours are associated with increased risk of DS only under reduced job satisfaction condition, which highlights the importance of improving job satisfaction, particularly among those working excessive hours.

## INTRODUCTION

Depression/depressive symptoms (DDS) is one of the most common and most costly mental health disorders, contributing to work impairment and reduced productivity affecting a large number of working people [1]. According to a report by the World Health Organization in 2012, it was estimated that 350 million people suffered from depression worldwide [2]. In Japan, nearly 2 million people were thought to have suffered from depression in 2005 [3]. A study that addressed the economic impact of depression in Japan estimated that the yearly cost was approximately $11 billion and of this cost 62.8% was work-related depression [4].

Long working hours and overtime have been suspected to be a critical risk factor for DDS, although the findings are not always consistent [5]. To my knowledge, there are a total of 33 studies that have examined the relationship between long working hours/overtime and DDS, and of these reports, 21 studies found significant positive associations [6–26], while 12 studies reported insignificant or even an inverse relationship between the two [27–38]. Inconsistent findings across studies may be due partly to differences in sample sizes, characteristic of study populations, definition of working hours/depression, analytic approaches, and covariates adjusted for, as well as study designs (leading to possible cause-effect reversals), as pointed out by some researchers [15, 18, 19, 35]. However, there are also other potentially important work-related factors that may have contributed to mixed results. It is becoming increasingly clear that the perception of psychosocial work conditions affect the relationship between work hours and psychological health [39–41]. For example, if employees are satisfied with or enjoying their job they may work long without suffering from DDS, whereas those who are dissatisfied with their job may not or could not work long because of depressed mood. Indeed, a meta-analysis on the relationship between job satisfaction and health confirmed that increased job satisfaction is protective against DDS [42]. Furthermore, a study that investigated the relationship between overtime and psychological health found that employees working overtime combined with low rewards had an elevated risk of poor recovery, burnout, negative home-to-work interference, and negative work-to-home interference compared to those with no overtime/high rewards (reference group), whereas those with overtime/high rewards did not show such an increase compared to the reference group [39].

Other work conditions that deserve attention are the influence of work schedule and status of employment. Regarding work schedule, a growing body of evidence suggests that night/rotating/irregular shift work is more harmful to mental health than daytime work condition [15, 37, 43–45], suggesting that the research on the relationship between work hours and DDS should rule out or at least statistically control for the influence of work schedules to produce more accurate estimates [5]. With respect to employment status, part-time employment has been reported to be more detrimental to health than full-time employment, especially among those involuntarily working part-time [32, 46, 47]. In accordance with these reports, one study confirmed the importance of distinguishing between overtime and long working hours among full- and part-time employees in relation to work-related outcomes, i.e., motivation and fatigue [48].

Based on these assumptions, the present study was designed to investigate the possible moderating role of job satisfaction in the relationship between working hours and depressive symptoms in full-time employees working under non-shift daytime condition. The aim of this study was to answer three research questions: 1) Is there an association between long working hours and depressive symptoms? 2) Is there an association between job satisfaction and depressive symptoms? 3) Do the combination of working hours and job satisfaction together relate to depressive symptoms? If so, which factor is mainly related to depressive symptoms? I address these questions using a sample of 2,375 full-time employees from 329 small and medium-scale businesses (SMBs) comprised of various industry sectors and occupations.

## RESULTS

Descriptive statistics for participants are shown in Table 1. Roughly 73% and 27% of participants were men and women, respectively. Overall, 45% of participants were aged 50 years and older, 68% married, 80% had high school education or higher, 48% current smoker, 68% alcohol drinker, 91% coffee/tea drinker, 38% slept less than 6 hrs/day, 21% had BMI 25 or higher, 76% had no physical/psychological symptoms, and 14% used medications. Regarding occupational factors, 43% worked in production/manufacturing, 44% metalworking, and 38% working in a business with employees less than 18 people.

**Table 1:** Sample descriptive statistics (*N* = 2,375)

Characteristics	*N*	(%)
**Total participants**	*2,375*	*(100)*
**Sociodemographic and socioeconomic factors**:		
Sex		
Men	*1,739*	*(73.2)*
Women	*636*	*(26.8)*
Age group, years		
18–29	*383*	*(16.1)*
30–39	*522*	*(22.0)*
40–49	*399*	*(16.8)*
50–59	*723*	*(30.4)*
60+	*348*	*(14.7)*
Marital status		
Married	*1,613*	*(67.9)*
Single	*605*	*(25.5)*
Separated/divorced/widowed	*157*	*(6.6)*
Educational level		
Junior high school	*486*	*(20.5)*
High school	*1,166*	*(49.1)*
Vocational/junior college	*336*	*(14.1)*
College/graduate school	*387*	*(16.3)*
**Health indicators**:		
Smoking status		
Lifetime nonsmoker	*987*	*(41.6)*
Former smoker	*240*	*(10.1)*
Current smoker (> 0 to ≤ 10 cigarettes/day)	*192*	*(8.1)*
Current smoker (> 10 to ≤ 20 cigarettes/day)	*639*	*(26.9)*
Current smoker (> 20 cigarettes/day)	*317*	*(13.3)*
Drinking habit		
Non-drinker	*757*	*(31.9)*
Occasional (> 0 to ≤ 3 times/week)	*578*	*(24.3)*
Frequent (≥ 4 times/week)	*1,040*	*(43.8)*
Caffeine intake (cups of coffee or tea/day)		
Almost none	*212*	*(8.9)*
1 to 2	*1,119*	*(47.1)*
3+	*1,044*	*(44.0)*
Sleep hours per day		
< 6	*912*	*(38.4)*
≥ 6	*1,463*	*(61.6)*
Body Mass Index		
< 20	*419*	*(17.6)*
≥ 20 to < 22.5	*772*	*(32.5)*
≥ 22.5 to < 25.0	*684*	*(28.8)*
≥ 25.0	*500*	*(21.1)*
Number of physical/psychological symptoms^a^		
None	*1,801*	*(75.8)*
1	*491*	*(20.7)*
2 or more	*83*	*(3.5)*
Use of medication^b^		
No	*2,035*	*(85.7)*
Yes	*340*	*(14.3)*
**Occupational factors**:		
Job type		
Managerial/clerical	*642*	*(27.0)*
Sales/service	*170*	*(7.2)*
Technical	*97*	*(4.1)*
Production/Manufacturing	*1,026*	*(43.2)*
Other	*440*	*(18.5)*
Industry sector		
Ceramic/clay/stone	*46*	*(1.9)*
Textile	*40*	*(1.7)*
Papermaking	*128*	*(5.4)*
Printing	*41*	*(1.7)*
Chemical	*308*	*(13.0)*
Leather	*15*	*(0.6)*
Metalworking	*1,033*	*(43.5)*
Food	*127*	*(5.3)*
Machinery	*376*	*(15.8)*
Other	*261*	*(11.0)*
Size of company by number of employees (in quintiles)		
1–8 workers	*412*	*(17.3)*
9–18 workers	*506*	*(21.3)*
19–31 workers	*495*	*(20.8)*
32–61 workers	*515*	*(21.7)*
62+ workers	*447*	*(18.8)*
Job control (in tertiles)		
High	*798*	*(33.6)*
Medium	*795*	*(33.5)*
Low	*782*	*(32.9)*
Quantitative workload (in tertiles)		
Low	*895*	*(37.7)*
Medium	*770*	*(32.4)*
High	*710*	*(29.9)*

^a^ Physical/psychological symptoms include hypertension, hyperlipidemia, diabetes mellitus, menopausal syndrome, cardiovascular disease, cancer, stomach/duodenal ulcer, arrhythmia, gout, hyperuricemia, renal disease, liver disease, stroke, gynecologic diseases, hyperthyrodism, peptic ulcer, severe allergy, hernia, back pain, rheumatoid arthritis, and panic disorder. ^b^Medications include aspirin, acetaminophen, β-blockers, cold/flu medicine, anti-hypertensives, naproxen, corticosteroids, and ibuprofen.

Overall prevalence and prevalence of depressive symptoms (CES-D ≥ 16) by working hours and job satisfaction are shown in Table 2. The prevalence of depressive symptoms among this population was 30.3% (95% CI 28.4–32.1). Working 12 hrs/day or more (compared to 6–8 hrs/day) and reduced job satisfaction were associated with increased depressive symptoms in a dose-response manner, but the strength of association with depressive symptoms seemed to be more pronounced for job satisfaction than for working hours. Prevalence of depressive symptoms among those who reported ‘very satisfied’ with their job had 16.8% while those reporting ‘somewhat satisfied,’ ‘not too satisfied,’ and ‘not at all satisfied’ had 27.6%, 36.0%, and 51.4%, respectively.

**Table 2:** Prevalence of depressive symptoms by working hours and job satisfaction (*N* = 2,375)

Variables	*N*	(%)	CES-D Score ≥ 16, % (95% CI)
**Overall prevalence**	*2,375*	*(100.0)*	30.3 (28.4 to 32.1)
**Working hours per day:^a^**			
6 to 8	*1,144*	*(48.2)*	28.4 (25.8 to 31.0)
9	*506*	*(21.3)*	28.9 (24.9 to 32.8)
10	*416*	*(17.5)*	31.5 (27.0 to 36.0)
11	*121*	*(5.1)*	37.2 (28.6 to 45.8)
12+	*188*	*(7.9)*	38.3 (31.3 to 45.2)
**Working hours per day:^b^**			
6 to 8	*1,144*	*(48.2)*	28.4 (25.8 to 31.0)
> 8 to 10	*922*	*(38.8)*	30.0 (27.1 to 33.0)
> 10	*309*	*(13.0)*	37.9 (32.5 to 43.3)
**Job satisfaction:^c^**			
Very satisfied	*280*	*(11.8)*	16.8 (12.4 to 21.2)
Somewhat satisfied	*1,306*	*(55.0)*	27.6 (25.2 to 30.1)
Not too satisfied	*614*	*(25.9)*	36.0 (32.2 to 39.8)
Not at all satisfied	*175*	*(7.4)*	51.4 (44.0 to 58.8)
**Job satisfaction:^c^**			
Very satisfied/Somewhat satisfied	*1,586*	*(66.8)*	25.7 (23.6 to 27.9)
Not too satisfied/Not at all satisfied	*789*	*(33.2)*	39.4 (36.0 to 42.8)

^a^ *p* < 0.05, ^b^*p* < 0.01, ^c^*p* < 0.001 (Chi-squared test).

Direct associations of working hours and job satisfaction with depressive symptoms as estimated by multivariable logistic regression analyses are shown in Table 3. Participants working 12+ hrs/day had significantly higher odds of depressive symptoms than those working 6 to 8 hrs/day (reference group) even after controlling for confounders. Furthermore, the trichotomized analysis found that participants working > 10 hrs/day had significantly increased odds of depressive symptoms than the reference category.

**Table 3:** Association of working hours and job satisfaction with depressive symptoms (*N* = 2,375)

	Model 1^a^	Model 2^b^	Model 3^c^	Model 4^d^
**Variables**	**OR (95% CI)**	**OR (95% CI)**	**OR (95% CI)**	**OR (95% CI)**
**Working hours per day**:				
6 to 8	1.00 (reference)	1.00 (reference)	1.00 (reference)	1.00 (reference)
9	1.02 (0.81 to 1.29)	1.04 (0.82 to 1.32)	1.03 (0.81 to 1.31)	1.09 (0.84 to 1.42)
10	1.16 (0.91 to 1.48)	1.25 (0.96 to 1.62)	1.20 (0.92 to 1.56)	1.16 (0.87 to 1.55)
11	1.49 (1.01 to 2.21)^e^	1.56 (1.04 to 2.35)^e^	1.45 (0.96 to 2.20)	1.51 (0.96 to 2.37)
12+	1.56 (1.14 to 2.16)^e^	1.64 (1.17 to 2.30)^e^	1.44 (1.02 to 2.04)^e^	1.49 (1.00 to 2.22)^e^
**Working hours per day**:				
6 to 8	1.00 (reference)	1.00 (reference)	1.00 (reference)	1.00 (reference)
> 8 to 10	1.08 (0.89 to 1.31)	1.12 (0.92 to 1.38)	1.10 (0.89 to 1.35)	1.12 (0.89 to 1.40)
> 10	1.54 (1.18 to 2.00)^f^	1.60 (1.20 to 2.13)^f^	1.43 (1.07 to 1.92)^e^	1.49 (1.07 to 2.08)^e^
**Job satisfaction**:				
Very satisfied	1.00 (reference)	1.00 (reference)	1.00 (reference)	1.00 (reference)
Somewhat satisfied	1.89 (1.35 to 2.65)^f^	1.79 (1.28 to 2.52)^f^	1.82 (1.29 to 2.56)^f^	2.04 (1.42 to 2.92)^f^
Not too satisfied	2.79 (1.96 to 3.97)^f^	2.52 (1.76 to 3.61)^f^	2.55 (1.78 to 3.67)^f^	2.92 (1.99 to 4.30)^f^
Not at all satisfied	5.25 (3.41 to 8.08)^f^	4.52 (2.92 to 7.01)^f^	4.50 (2.90 to 7.00)^f^	5.51 (3.41 to 8.89)^f^
**Job satisfaction**:				
Very satisfied/Somewhat satisfied (high)	1.00 (reference)	1.00 (reference)	1.00 (reference)	1.00 (reference)
Not too satisfied/Not at all satisfied (low)	1.88 (1.57 to 2.25)^f^	1.75 (1.46 to 2.11)^f^	1.75 (1.45 to 2.11)^f^	1.81 (1.47 to 2.22)^f^

^a^ Unadjusted.

^b^ Adjusted for sex, age group, marital status, and educational level.^c^ Adjusted for sex, age group, marital status, educational level, smoking, drinking, caffeine intake, sleep duration, and BMI.^d^ Adjusted for sex, age group, marital status, educational level, smoking, drinking, caffeine intake, sleep duration, BMI, number of physical/psychological symptoms, use of medication (yes/no), job type, industry sector, company size, job control (high, medium, low), and male/female ratio.^e^ *p* < 0.05, ^f^ *p* < 0.001.

Regarding job satisfaction, participants reporting ‘not at all satisfied,’ ‘not too satisfied,’ and ‘somewhat satisfied’ had significantly increased odds of depressive symptoms compared to those reporting ‘very satisfied’ with their job (reference group). The dichotomized analysis found that participants reporting low job satisfaction had 75% to 88% increase of depressive symptoms than those with high satisfaction.

**Figure 1:**
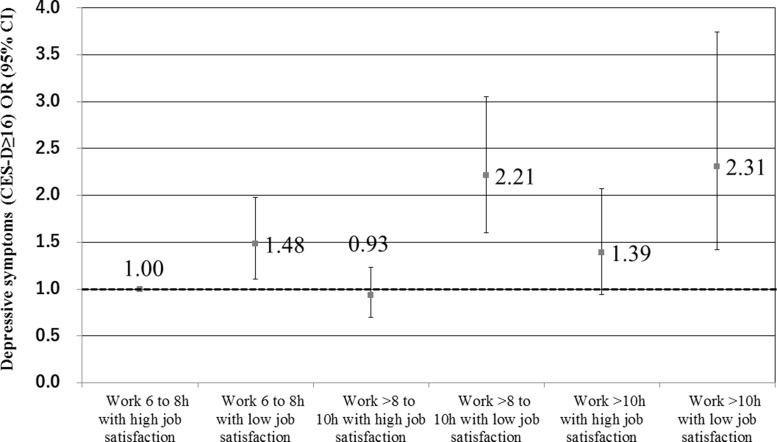
Combined association of working hours and job satisfaction on depressive symptoms

The combined association of working hours and job satisfaction with depressive symptoms are shown in Table 4 and Figure 1. As compared with a reference group that had a 6 to 8 hrs/day working hours with high job satisfaction, the odds of depressive symptoms were significantly higher among participants working 6 to 8 hrs/day, > 8 to 10 hrs/day or > 10 hrs/day with low job satisfaction. Although participants working > 10 hrs/day with high job satisfaction had increased depressive symptoms compared with the reference group in models 1 and 2 (*p <* 0.05), the significance disappeared after further adjustment for additional covariates (models 3 and 4).

**Table 4:** Combined association of working hours and job satisfaction with depressive symptoms (*N* = 2,375)

			Model 1^a^	Model 2^b^	Model 3^c^	Model 4^d^
**Variables**	***N***	***(%)***	**OR (95% CI)**	**OR (95% CI)**	**OR (95% CI)**	**OR (95% CI)**
**Working hours and job satisfaction**:						
**Working 6 to 8 h/day with high job satisfaction**	*768*	*(32.3)*	1.00 (re^f^erence)	1.00 (re^f^erence)	1.00 (re^f^erence)	1.00 (re^f^erence)
Working 6 to 8 h/day with low job satisfaction	*376*	*(15.8)*	1.58 (1.21 to 2.07)^g^	1.48 (1.13 to 1.94)^f^	1.47 (1.12 to 1.93)^f^	1.48 (1.11 to 1.98)^f^
Working > 8 to 10 h/day with high job satisfaction	*618*	*(26.0)*	0.92 (0.72 to 1.18)	0.97 (0.75 to 1.25)	0.93 (0.72 to 1.21)	0.93 (0.70 to 1.23)
Working > 8 to 10 h/day with low job satisfaction	*304*	*(12.8)*	2.21 (1.67 to 2.92)^g^	2.16 (1.61 to 2.89)^g^	2.13 (1.59 to 2.86)^g^	2.21 (1.60 to 3.05)^g^
Working more than 10 h/day with high job satisfaction	*200*	*(8.4)*	1.49 (1.07 to 2.09)e	1.56 (1.09 to 2.22)e	1.41 (0.98 to 2.20)	1.39 (0.94 to 2.07)
Working more than 10 h/day with low job satisfaction	*109*	*(4.6)*	2.51 (1.66 to 3.78)^g^	2.45 (1.59 to 3.76)^g^	2.13 (1.38 to 3.30)^g^	2.31 (1.42 to 3.74)^g^

^a^ Unadjusted.

^b^ Adjusted for sex, age group, marital status, and educational level.

^c^ Adjusted for sex, age group, marital status, educational level, smoking, drinking, caffeine intake, sleep duration, and BMI.

^d^ Adjusted for sex, age group, marital status, educational level, smoking, drinking, caffeine intake, sleep duration, BMI, number of physical/psychological symptoms, use of medication (yes/no), job type, industry sector, company size, job control (high, medium, low), and male/female ratio.

^e^ *p* < 0.05, ^f^ *p* < 0.01, ^g^ *p* < 0.001.

## DISCUSSION

The purpose of this study was to investigate the independent and combined association of working hours and job satisfaction with depressive symptoms in a large number of SMB employees in a suburb of Tokyo. The current study produced three main findings. First, long working hours, particularly those working 12+ hrs/day (compared to those working 6 to 8 hrs/day), were weakly but significantly associated with increased risk of depressive symptoms. Second, reduced job satisfaction was strongly related to depressive symptoms in a dose-response manner. Third and most importantly, the combination of working hours with job satisfaction had a synergistic association with depressive symptoms, but this association was observed only when different working hours were combined with low job satisfaction (compared to those working 6–8 hours/day with high satisfaction). Although the results of this study should be interpreted with caution in light of self-reporting and cross-sectional design, these data imply that job redesign/crafting aimed at enhancing job satisfaction may prevent, or at least reduce, occurrence of workplace DDS associated with long working hours.

Data from past studies have suggested that long working hours are associated with increased risk of DDS [6–26]. At the same time, there are also studies that reported no significant relationship [27–36] or even an inverse relationship between the two [37, 38]. As stated earlier in the Introduction, several plausible explanations have been proposed for its inconsistent findings. In addition to these explanations, this study yielded an alternative explanation as to why work hour-DDS relationship is not simple or straightforward. The results of this study suggested that long working hours do not necessarily have negative psychological health consequences if employees are working under favorable/positive working condition, i.e., high job satisfaction. Conversely, the risk of DDS associated with long working hours are more pronounced if employees are working under poor/negative condition, i.e., low job satisfaction. The finding is supported by several studies that considered working condition in relation to long work hours and health [39, 40]. One study reported that long work hours are not necessarily related to adverse psychological health if job rewards are high, even under high pressure to work overtime among postal service employees [39]. Another study observed a positive association between weekly working hours and poor physical health among train drivers who worked overtime with high pressure and low social support, whereas those under high pressure with high social support yielded an inverse relationship [40]. These findings, together with the current results, support the notion that psychosocial work condition could serve as a moderator in the relationship between working hours and health.

Interestingly, those who worked moderately long hours (> 8 to 10 hrs/day) with high job satisfaction had the lowest risk of depressive symptoms compared to those working 6–8 hrs/day with high job satisfaction (aOR=0.92 to 0.97). Although the results only yielded a small difference, it seems reasonable to think that this population is consisted of healthiest group of employees who are working voluntarily with high motivation. Two studies from Netherlands suggested that moderate overtime is not always harmful to health outcomes [49, 50]. For instance, a study using a representative sample of a Dutch full-time workforce found that voluntary overtime workers were non-fatigued and satisfied with their job even without rewards while involuntary overtime workers exhibited high fatigue level and less satisfaction [49].

More than a half of the participants who were ‘not at all satisfied’ with their job had depressive symptoms. This finding is in line with several empirical researches [51, 52] as well as a result of meta-analysis based on 485 studies of job satisfaction and health which reported that workers with low levels of satisfaction were more likely to experience DDS (*ρ* =.428) [42], indicating that job satisfaction is an important predictor of psychological health. Therefore, those with extremely low levels of job satisfaction may need an immediate care to prevent workplace depression.

In this study, 30.3% of participants had CES-D scores of 16 or higher. The prevalence is similar to several studies using same criteria among the working populations (ranging from 24.5% to 33.9%) [53–55]. In contrast, an estimate based on diagnostic criteria are often much lower. For example, a median 12-month prevalence of major depressive disorder based on 42 different studies yielded 5.3% with an interquartile range of 3.6% to 6.5% [56]. Similarly, lifetime and 12-month prevalence of major depressive disorder was reported to be 6.1% and 2.2%, respectively, based on the World Mental Health Japan Survey [57]. The prevalence gap between the former and latter studies could be attributable to different criteria for defining DDS, i.e., questionnaire vs. diagnostic criteria. In consideration of these facts, studies using both methodology/definition simultaneously may help understand the relationship between long working hour and DDS more precisely.

### Strengths and limitations

A principal strength of this study is that it not only explored the independent association of working hours and job satisfaction with depression but also examined the combined associations of working hours and job satisfaction on depressive symptoms in a fairly large number of full-time employees of SMBs representing various industry sectors and occupations. Furthermore, participants under shift work and non-full-time condition, who reported major depressive disorders and anxiety disorders, as well as those working < 6 h/day and > 20 h/day were excluded to minimize selection bias leading to under- or overestimation. The limitations of this study are as follows. First, since this is a cross-sectional data, the association could be in either direction, i.e., long work hours and diminished job satisfaction may increase the risk of depressive symptoms or that undiagnosed depression or depressive personality traits may be the cause for reduced job satisfaction and short working hours. Second, work hours, job satisfaction, and depressive symptoms were assessed by self-report rather than through the use of objective measures or diagnostic criteria. Third, response bias may have occurred if non-respondents differed from respondents; in particular, those who worked extremely long hours may have had less time available to respond to the questionnaire. Fourth, although the study included a fairly large number of confounders, information on unmeasured work-related factors such as work-family conflict and organizational justice and non-work-related variables such as personality traits and genetic components, as well as unknown common factors for both depressive symptoms and job satisfaction were not included in the analyses.

## MATERIALS AND METHODS

### Study participants and procedure

The study design was cross-sectional and data were collected using a self-administered questionnaire between August and December 2002. The study sample consisted of full-time employees of SMBs in a size ranging from 1 to 158 workers in the city of Yashio, Saitama, and in the Ohta ward of Tokyo. Yashio has the highest percentage of manufacturing plants in Saitama prefecture. The ward of Ohta, which is a so-called “industrial area,” is unique for its number of SMBs. About 20% of SMBs in both areas were selected weighted by distribution of industry sector types, resulting in 329 SMBs from Yashio and 61 from the Ohta ward. An occupational health nurse/physician contacted each representative of the company to request participation in the questionnaire survey. Among these businesses, 248 in Yashio and 52 in Ohta agreed to participate. Questionnaires were distributed during visits to each business and were given to 2,591 employees in Yashio and 1,102 employees in Ohta (*n* = 3,693). Finally, responses were obtained from 2,884 employees (2,022 men and 862 women) from 296 businesses (response rate 78.1%). Those who had missing responses to sex, age, working hours, and job satisfaction were eliminated from the analyses (*n* = 126). Similarly, those who had 6 or more missing responses on the Center for Epidemiologic Studies Depression Scale (CES-D) (see ‘Measurements’ section for detail) and those who had been diagnosed with major depressive disorder or anxiety disorders were excluded from the analysis (*n=* 64). In addition, those who reported working < 6 hrs/day or > 20 hrs/day, working under non-day shifts or < 18 years old were excluded (*n* = 131). Since there were less than 5% missing responses for all the covariates in this study, Missing Value Analysis was performed using IBM SPSS Statistics 21.0 software (SPSS, Inc., Chicago, IL, USA) [58]. The ‘expectation–maximization method’ of imputing missing values was utilized. As a result, following variables, i.e., marital status, educational level, smoking status, drinking habit, caffeine intake, sleep hours, BMI, number of physical/psychological symptoms, use of medication, job type, job control, and quantitative workload were imputed. Thus, data on a total of 2,375 participants (1,739 men and 636 women) working under non-shift daytime condition were used in the final analyses. The study was approved by the Medical Ethical Committee of the University of Tokyo. All procedures followed were in accordance with the ethical standards of the responsible committee on human experimentation (institutional and national) and with the Helsinki Declaration of 1975, as revised in 2000. Informed consent was obtained from all participants for being included in the studies presented.

### Variables

#### Working hours

Working hours were determined by an open-ended question: How many hours do you usually work in a typical working day? Number of hours were grouped into three categories (i) 6 to 8 hrs/day, (ii) > 8 to 10 hrs/day, and (iii) > 10 hrs/day.

### Job satisfaction

Job satisfaction was assessed by a single-item assessment tool included in the Japanese version of the generic job stress questionnaire (GJSQ) developed by the U.S. National Institute for Occupational Safety and Health (NIOSH) which is a well-established means of measurement [59–61]. Item/response for the scale is as follows: All in all, how satisfied would you say you are with your job? (1) not at all satisfied, (2) not too satisfied, (3) somewhat satisfied, (4) very satisfied. The item has been frequently used in past studies to measure job satisfaction at the workplaces [51]. Job satisfaction was dichotomized into low (not at all satisfied/not too satisfied) and high (somewhat satisfied/very satisfied) levels.

### Depressive symptoms

Depressive symptoms was measured using a Japanese version of the Center for Epidemiologic Studies Depression scale (CES-D) [62]. The 20-item depressive symptom scale measures the level of depressive symptoms experienced in the past week. The CES-D scale cut-off score is 16, which differentiates between those exhibiting high levels of depressive symptoms (score ≥ 16) and those with lower levels of such symptoms (score < 16) [63]. The internal consistency of the CES-D scale for the study sample was 0.84.

### Covariates

Covariates considered included sociodemographic and socioeconomic factors, health behaviors, biological factors, medication usage, and occupational factors as listed in Table 1. Daily sleep hours during the previous 1-year period were assessed by a following questionnaire: On average, how much sleep at night do you usually get? Response options were: < 5 hrs/5 to < 6 hrs/6 to < 7 hrs/7 to < 8 hrs/8 to < 9 hrs/9+ hrs. A previous study confirmed a strong convergent and discriminant validity as well as a high level of test-retest stability over 1 year for this question [64]. Information on height and weight were obtained to assess body mass index (BMI), calculated as weight (kg) divided by height (m) squared, and divided into four groups. Job control and quantitative workload were evaluated by the Japanese version of the NIOSH GJSQ. Job control measures how much the worker feels that tasks, workplace setting, and decisions at work are controllable and is assessed based on 16-items, while quantitative workload estimates how much work must be done on daily basis and is based on 4-items. Internal consistency (Cronbach's alpha) for these scales was 0.96 and 0.88, respectively.

Participants were asked if they were treated for any of the following disorders or symptoms: hypertension, hyperlipidemia, diabetes mellitus, major depressive disorder, menopausal syndrome, or other. If the participants reported ‘other disorders,’ they were asked to specify the condition. Participants reported various disorders as listed on the bottom of Table 1. The numbers of disorders among the participants were counted and were included as a covariate.

### Statistical analyses

Prevalence of depression by working hours and job satisfaction was analyzed by Chi-squared test. The risk of depression by working hours and job satisfaction was estimated by multivariable logistic regression with odds ratios (ORs) and 95% confidence intervals (CIs) as measures of association. Combined associations of working hours and job satisfaction were examined by a similar analytic method. They were divided into six groups as follows: three groups of working hours (< 6 hrs/day, 6 to < 8 hrs/day, or 8+ hrs/day) × two groups of job satisfaction (low versus high). The interactive associations of working hours and job satisfaction on depression were also examined. Adjustments for covariates were made in a stepwise fashion. A crude OR was computed in Model 1. The second model included sociodemographic and socioeconomic factors as covariates (Model 2). The third model included health behaviors and biological factors in addition to model 2 covariates (Model 3). And finally, occupational factors were included in addition to model 3 covariates (Model 4). Quantitative workload was left out of multivariable logistic regression analyses because of a strong intercorrrelation with working hours and some recent studies indicated that work demands should be treated as an intermediate variable but not as a confounder [17, 22]. The significance level for all statistical analyses was *P* < 0.05 (two-tailed test). Data were analyzed using IBM SPSS version 21.0 software (SPSS, Inc., Chicago, IL, USA).

## CONCLUSIONS

This study found independent associations of working hours and job satisfaction with depressive symptoms. However, when the combined associations of working hours and job satisfaction were tested, job satisfaction turned out to be the main factor related to depressive symptoms. Furthermore, a combination of long working hours with reduced job satisfaction exerted a reciprocal association on depressive symptoms, but a combination of long working hours with high satisfaction did not show such an effect. Prospective research is warranted to determine the causal mechanisms underlying the present findings.

## Abbreviations

DDS: depression/depressive symptoms
CES-D: Center for Epidemiologic Studies Depression scale
SMB: small and medium-scale business
NIOSH: National Institute for Occupational Safety and Health
GJSQ: generic job stress questionnaire
OR: odds ratio
aOR: adjusted odds ratio
CIs: confidence intervals
BMI: body mass index
